# Computational cell fate modelling for discovery of rewiring in apoptotic network for enhanced cancer drug sensitivity

**DOI:** 10.1186/1752-0509-9-S1-S4

**Published:** 2015-01-21

**Authors:** Shital K Mishra, Sourav S Bhowmick, Huey Eng Chua, Fan Zhang, Jie Zheng

**Affiliations:** 1 School of Computer Engineering, Nanyang Technological University, Singapore; 2 Genome Institute of Singapore (GIS), Biopolis, Singapore

## Abstract

The ongoing cancer research has shown that malignant tumour cells have highly disrupted signalling transduction pathways. In cancer cells, signalling pathways are altered to satisfy the demands of continuous proliferation and survival. The changes in signalling pathways supporting uncontrolled cell growth, termed as rewiring, can lead to dysregulation of cell fates e.g. apoptosis. Hence comparative analysis of normal and oncogenic signal transduction pathways may provide insights into mechanisms of cancer drug-resistance and facilitate the discovery of novel and effective anti-cancer therapies. Here we propose a hybrid modelling approach based on ordinary differential equation (ODE) and machine learning to map network rewiring in the apoptotic pathways that may be responsible for the increase of drug sensitivity of tumour cells in triple-negative breast cancer. Our method employs Genetic Algorithm to search for the most likely network topologies by iteratively generating simulated protein phosphorylation data using ODEs and the rewired network and then fitting the simulated data with real data of cancer signalling and cell fate. Most of our predictions are consistent with experimental evidence from literature. Combining the strengths of knowledge-driven and data-driven approaches, our hybrid model can help uncover molecular mechanisms of cancer cell fate at systems level.

## Introduction

The objective of anti-cancer therapeutics is to kill cancer cells with minimum damage to the healthy cells. To this end, a solid understanding of the cell fate decisions (*e.g*. apoptosis, proliferation) of different cells under various conditions would be required. It is well known that signalling pathways play crucial roles in the regulation of cancer cell fate [1]. However, it is challenging to understand the dynamics of signal transduction at systems level, due to non-linearity of the network dynamics, *e.g*. feedback and crosstalk. In cancer cells, this becomes even more complicated due to various types of alterations (*e.g*. DNA mutations, genome rearrangement, epigenetic changes, and pathway alterations). These alterations allow cancer cells to adapt to new conditions and evolve drug resistance. Therefore, to find effective anti-cancer therapies, cancer-specific alterations in the signalling pathways must be taken into account. Moreover, it is desirable to understand how cancer cells respond to different combinations of drugs, and how drug sensitivity can be enhanced. Genomic and proteomic data of cellular responses to drugs, synchronized with cell fate observations, would shed light on cancer drug effects at systems level. However, even if sufficient data are available, it is challenging to construct a model of cell signalling to explain the data and make accurate predictions. As more "omics" data about cancer are available recently, computational methods for modelling and discovery of cancer cell fate are becoming more important.

Cancer cell fate in response to drugs has been studied with both discrete and continuous models using knowledge driven approaches. For example, the study in [2] focused on discrete modelling of the apoptosis network, by constructing a model for cell fate with 25 key regulatory genes (*e.g*. Casp3, BCL2, XIAP, etc.). Cell survival pathways with cell death (necrosis and apoptosis) pathway were combined to model three cell fates, namely apoptosis, proliferation and survival. GINsim [3] software was used to perform simulation to assess the importance of Cytokines (*e.g*. TNF, FASL) in deciding the cell fate. In another study, Hong *et al*. [4] analysed the continuous model of apoptosis triggered by the drug Cisplatin. They initially constructed apoptosis pathways using existing literature. This model was configured to respond to the external stimulus, *i.e*. the drug Cisplatin. To analyse the functions of various signalling pathways and their crosstalk, Hong *et al*. integrated three apoptosis pathways, namely death receptors (*e.g*. FasL) induced pathway, mitochondrial pathway and ER stress pathway. Using their differential equation based model they found that the apoptosis caused by Cisplatin was dependent on doses and time. The level of apoptosis was almost stagnant at higher concentration of drug. They also observed that mitochondrial pathway has strongest effect on apoptosis (among the three pathways) [4].

On the other hand, data driven modelling of signalling pathways is a promising approach to uncover regulatory mechanism for cancer cell fate. The study in [5] investigated three lines of breast tumour, namely BT-20, MDA-MB-453 and MCF7, for their responses to various combinations of exposure to several genotoxic drugs and signalling inhibitors. The authors found that it was the pre-treatment, rather than co-treatment or post-treatment, of a subset of TNBCs with Epidermal Growth Factor Receptor (EGFR) inhibitors that can enhance the sensitivity of tumour cells in their apoptosis response to DNA-damaging chemotherapy. The study suggested that such a treatment may lead to the rewiring of oncogenic signalling pathways which has the potential to make cancer cells more susceptible to death. It was further reported that the inhibition of EGFR in a time-staggered way may be responsible for sensitising tumour cells to genotoxic drugs. However, simultaneous co-administration of inhibitors and genotoxic drug could not make the tumour cells less tumorigenic. To test these hypotheses, the study systematically investigated a series of drug combinations for their effects on breast cancer cells. The study suggests that rewiring inside the tumour cells is responsible for the increase of drug sensitivity, but it is not clear where and how the rewiring happens. The rewiring involves alterations of signalling pathways such as addition or deletion of edges in the network, change in reactions rates, and change in the concentration of molecules. Since it is more challenging to directly observe rewiring signalling pathways by wet lab experiments, computational methods for predicting rewiring from data would be useful for study of cancer drug effect and cell fate decisions.

In this paper, we propose a hybrid modelling approach that combines the advantages of knowledge-driven and data-driven approaches for modelling cancer signalling pathways and cell fate decisions. From a generic apoptotic network, we simulate the time series data of signalling proteins using ODEs based on a network candidate modified from the generic network by *in silico *rewiring; then the simulated data are compared with the real data. This in *in silico *rewiring, simulation and data fitting is repeated iteratively to improve the goodness of fit using Genetic Algorithm. Through such an optimisation we can detect the topology alterations of the network that allows close fitting of model to the real data. As such, network rewiring can be inferred from real data. Most of rewiring events predicted by our model are supported by existing literature.

## Methods

Our work is broadly organised in three interrelated parts. We initially constructed a basic apoptosis network (say *N*_1_) which is free of abnormal mutation and represents signalling pathways inside a healthy cell. We used the biological data for our studies based on the phosphorylation levels of signalling proteins and phenotypic responses taken from the biological experiments conducted in [5]. Hereafter, we call this data set as "Yaffe's data". We compared the simulation data from this network with the Yaffe's data using Genetic Algorithm and Dynamic Time Warping for DMSO treatment and derived a tumorigenic network (say *N*_2_). Then we modified *N*_2 _to fit with Yaffe's data to derive a drug sensitive network (say *N*_3_). *N*_1 _was constructed directly from literature; *N*_2 _consisted of edges added and deleted from *N*_1 _by Genetic Algorithm; *N*_3 _consisted of edges added and deleted from *N*_2 _by Genetic Algorithm.

### Data

The Yaffe's data set contains six types of cellular phenotypic responses from three cancer cell lines, namely triple negative BT-20, Hormone sensitive MCF-7 and HER2 overexpressing MDA-MB-453. The six phenotypes are Apoptosis, Proliferation, G1, S, G2 and M. These phenotypic responses are linked with on 35 signalling proteins (only 32 available in the published data set). The signalling molecule data available contains 8 time points (0, 1, 2, 3, 4, 5, 6 and 7). The phenotypic responses were measured at five time points (0, 6, 7, 8 and 9), over 24 hours in the panel of breast cancer cell lines. The cell fates and signalling data are available for all the three cell lines. The measurements of the signalling and cell fate data have been done for six treatments namely DMSO, TAR, DOX, DT, T-D and D-T. The apoptosis level is reportedly high for the T-D treatment, meaning the cells are now less tumorigenic for this treatment and thereby more susceptible to death induced by DNA damage response pathways. The study in [5] employs several mathematical modelling approaches to relate signalling data to cell phenotypes. Principal component analysis (PCA) was used to identify co-variation between signals, whereas partial least-squares regression (PLSR) was used to identify co-variation between molecular signals and corresponding cellular phenotypic responses. In both PCA and PLSR modelling, the input vectors contained quantitative measurements of the signalling proteins.

### Apoptosis network construction

We constructed a generic apoptosis network consisting of 22 signalling proteins including 18 proteins from the biological experiment conducted in [5]. The authors of [5] provided Variable Importance in Projection (VIP) scores for different selected proteins to demonstrate how the selected proteins were useful for predicting apoptosis of different cell lines. For example, proteins with VIP score > 1 were important variables, proteins with VIP score < 0.5 were unimportant variables. Since our goal is to test the goodness of fit of our model with Yaffe's data, we selected proteins that have been measured in the experiment. Also, our signalling network includes molecules based on the prior knowledge from several pathways playing critical roles in inducing cell death, which include DNA damage response and cell death pathways, stress response pathways, etc.

**Figure 1:**
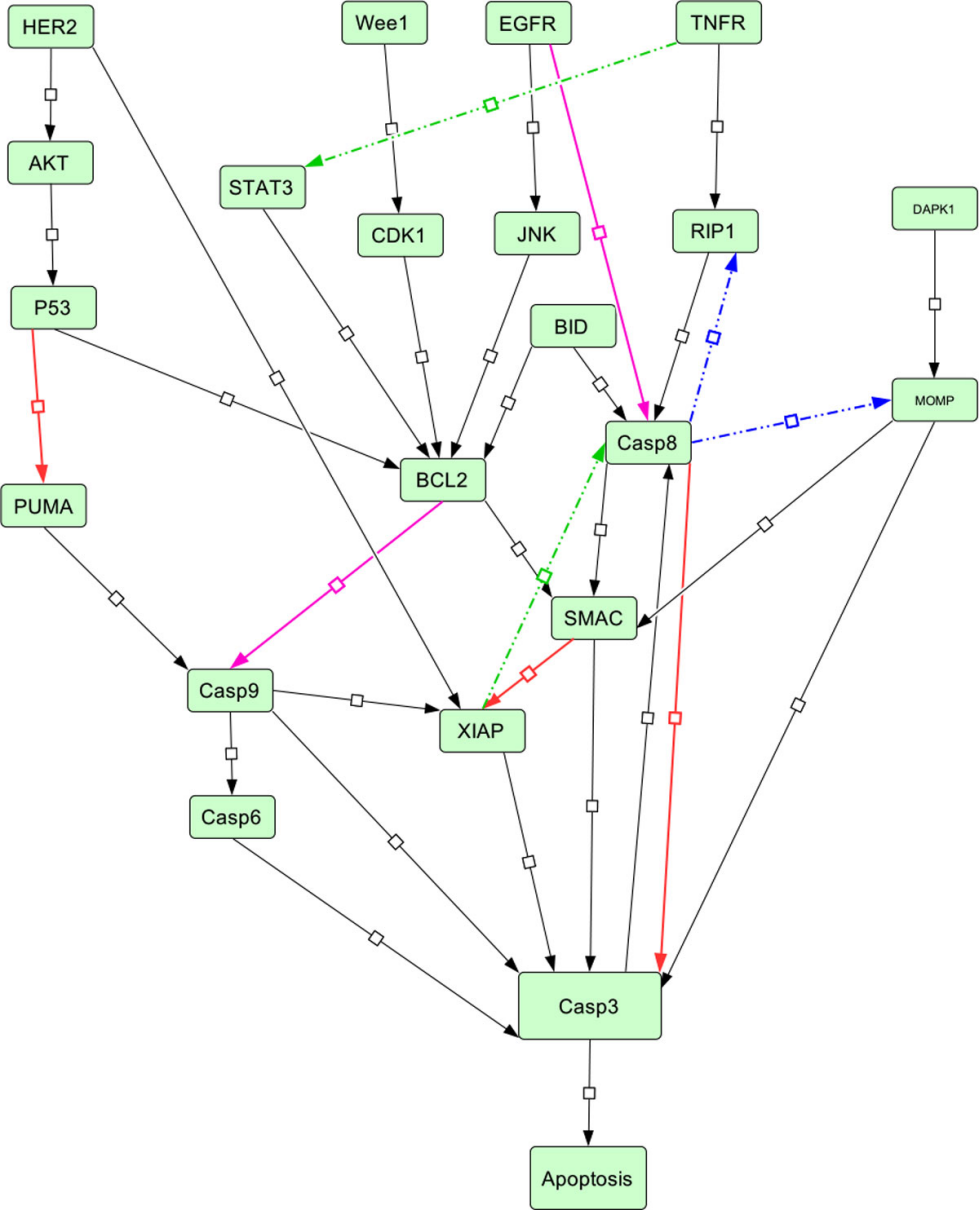
**Basic apoptosis network and various rewiring events**. Basic apoptotic network contained all edges except the broken edges (green and blue). Edges represented by thick red colour were deleted by Genetic Algorithm while inferring *N*_2 _from *N*_1_. Broken edges represented by green colour were inserted by Genetic Algorithm while inferring *N*_2 _from *N*_1_. Edges represented by magenta colour were deleted by Genetic Algorithm while inferring *N*_3 _from *N*_2_. Broken edges represented by blue colour were inserted by Genetic Algorithm while inferring *N*_3 _from *N*_2_.

The network diagram is shown in Figure 1. We constructed connections between various signalling proteins in our basic apoptosis network using the existing literature. HER2 is a cell transmembrane receptor which plays a significant role in breast cancer by suppression of tumour growth. It provides growth signals in PI3KAKT and Ras-MAPK pathways. HER2 also suppresses p53-mediated apoptosis by upregulation of MDM2 by activation of AKT. p53 also participates in promoting apoptosis by sensing DNA damage and can upregulate pro-apoptotic Bcl-2 proteins (*e.g*. PUMA) as well as suppress IAPs (*e.g*. survivin) [6]. PUMA is a pro-apoptotic protein and plays crucial roles in apoptosis. Tumour suppressor gene p53 regulates the expression of PUMA. Once activated, PUMA frees the apoptosis regulator Bax and Bak owing to its high affinity with Bcl-2 oncogenic group of proteins. The freed proteins further transduce the apoptotic signals to the mitochondria. This causes the Caspases to be activated and the activated caspases eventually lead to the cell death [7]. Activation of pro-apoptotic BAX/Bak facilitates heme protein cytochrome c being released from the inner mitochondria membrane [8,9]. The cytochrome c further stimulates Casp9, which then stimulates Casp3 and Casp7, which eventually lead to apoptotic cell death [10]. Up-regulation of DAPK induces apoptosis by enhancing response of tumour suppressor p53 whereas down-regulation of DAPK reduces the response of p53 to several oncogenes including c-Myc and E2F-1. The activated form of DAPK further activates the Beclin-1 by phosphorylation. Beclin-1 activation is crucial for Autophagy and also for its crosstalk with apoptosis.

Beclin-1 is an autophagy protein that interacts with Bcl-2, an anti-apoptotic protein, and they form a complex, to facilitate a switch from autophagy to apoptosis and vice-versa. The activity of Beclin-1, owing to the complex formation of Bcl-2:Beclin-1 complex, is suppressed by Bcl-2 protein [11,12]. STAT3 is an important gene due to its roles in regulating cell fate including cell survival and cell growth. Experimental results have shown that a decreased level of STAT3 leads to a reduced level of Bcl-2 [13]. Activation of STAT3 gene stimulates transcription of quite a few cell fate controlling genes, which include some of the members of Bcl-2 protein family [14]. The proteins Wee1 and Cdk1 (Cyclin-dependent kinase 1) play very important roles in regulating cell cycle. During mitosis, the size of a cell is controlled by the inhibition of Cdk1 by Wee1 which prevents the cell from going into mitosis. The suppression of Cdk1 by Wee1 is done by phosphorylation of Cdk1 [15]. Cdk1 in turn phosphorylates Bcl-2 and suppresses its activity [16]. The EGF receptor (EGFR) is a cell membrane protein receptor with high affinity for several specific proteins (ligands) including Epidermal Growth Factor (EGF). EGFR further activates many targets including Jun N-terminal kinases (JNKs). Mutation in EGFR may lead to its uncontrolled expression which can potentially cause uncontrolled cell growth, which is a hall mark of cancer [17]. The Bcl-2-associated death promoter (BAD), another member protein from Bcl-2 group of proteins, is highly pro-apoptotic. JNK can inhibit apoptosis by phosphorylating BAD protein [18]. Trimeric Tumor Necrosis Factor (TNF) activates multiple signalling pathways which in turn lead to apoptosis. TNF interacts with death domain (DD) protein TRADD. TRADD in turn has been shown to have high affinity for RIP [19]. Casp3 mediates cleavage of multiple proteins and causes DNA fragmentation which eventually leads to apoptosis [2].

### Simulation with ODE

Using the topology of the basic apoptosis network (Figure 1), constructed from the literature, we derived a computational model by generating rate equations for the network. The complete set of differential equations for the basic network *N*_1 _is given in Table 2. Concentration of each molecule is represented by enclosing the molecule name with square brackets (e.g. [Casp8]). Reaction rate constants are represented as *rc*_1_, *rc*_2 _etc. For example, based on the basic network *N*_1 _in Figure 1, the differential equations for the change of concentration of AKT and p53 were derived as:

**Table 2:** Differential equations for the basic apoptosis network *N*_1 _(Figure 1)

**S.N**.	Rate of Change	Differential equations
1	$\frac{d}{dt}\text{Casp}6$	- [Casp6]·*rc*_11 _+ [Casp9]·*rc*_12_
2	$\frac{d}{dt}\text{CDK}1$	+[Wee1]·*rc*_13 _- [CDK1]·*rc*_14_
3	$\frac{d}{dt}\text{Wee}1$	- [Wee1]·*rc*_13_
4	$\frac{d}{dt}\text{JNK}$	- [JNK]·*rc*_15 _+ [EGFR]·*rc*_17_
5	$\frac{d}{dt}\text{BID}$	- [BID]·*rc*_16 _- [BID]·*rc*_25_
6	$\frac{d}{dt}\text{EGFR}$	- [EGFR]·*rc*_17 _- [EGFR]·*rc*_32_;
7	$\frac{d}{dt}\text{PUMA}$	- [PUMA]·*rc*_19 _+ [p53]·*rc*_27_
8	$\frac{d}{dt}\text{Casp}3$	- [Casp3]·*rc*_1 _+ [XIAP]·*rc*_2 _+ [SMAC]·*rc*_4 _+ [Casp8]·*rc*_7 _- [Casp3]·*rc*_8 _+[Casp9]·*rc*_9 _+[Casp6]·*rc*_11 _+ [MOMP]·*rc*_30_
9	$\frac{d}{dt}\text{Casp9}$	- [Casp9]·*rc*_9 _- [Casp9]·*rc*_12 _+ [PUMA]·*rc*_19 _- [Casp9]·*rc*_24 _+ [BCL2]·*rc*_26_
10	$\frac{d}{dt}\text{HER2}$	- [HER2]·*rc*_20 _- [HER2]·*rc*_31_
11	$\frac{d}{dt}\text{Casp8}$	- [Casp8]·*rc*_3 _+ [RIP1]·*rc*_6 _- [Casp8]·*rc*_7 _+ [Casp3]·*rc*_8 _+ [BID]·*rc*_25 _+[EGFR]·*rc*
12	$\frac{d}{dt}\text{Apop}$	+[Casp3]·*rc*_1_
13	$\frac{d}{dt}\text{XIAP}$	- [XIAP]·*rc*_2 _+ [SMAC]·*rc*2_1 _+ [Casp9]·*rc*_24 _+ [HER2]·*rc*_31_
14	$\frac{d}{dt}\text{SMAC}$	+[Casp8]·*rc*_3 _- [SMAC]·*rc*_4 _+ [BCL2]·*rc*_5 _- [SMAC]·*rc*_21 _+ [MOMP]·*rc*_29_
15	$\frac{d}{dt}\text{BCL2}$	- [BCL2]·*rc*_5 _+ [p53]·*rc*_10 _+ [CDK1]·*rc*_14 _+ [JNK]·*rc*_15 _+[BID]·*rc*_16 _+[STAT3]·*rc*_22 _- [BCL2]·*rc*_26_
16	$\frac{d}{dt}\text{RIP1}$	- [RIP1]·*rc*_6 _+ [TNFR]·*rc*_23_
17	$\frac{d}{dt}\text{P53}$	- [p53]·*rc*_10 _+ [AKT]·*rc*_18 _- [p53]·*rc*_27_
18	$\frac{d}{dt}\text{AKT}$	- [AKT]·*rc*_18 _+ [HER2]·*rc*_20_
19	$\frac{d}{dt}\text{STAT3}$	- [STAT3]·*rc*_22_
20	$\frac{d}{dt}\text{TNFR}$	- [TNFR]·*rc*_23_
21	$\frac{d}{dt}\text{DAPK}1$	- [DAPK1]·*rc*_28_
22	$\frac{d}{dt}\text{MOMP}$	+[DAPK1]·*rc*_28 _- [MOMP]·*rc*_29 _- [MOMP]·*rc*_30_

$$\begin{array}{c} \frac{d}{dt}AKT=-\left[AKT\right]\cdot rc_{18}+\left[HER2\right]\cdot rc_{20},\\ \frac{d}{dt}p53=-\left[p53\right]\cdot rc_{10}+\left[AKT\right]\cdot |rc_{18}-\left[p53\right]\cdot rc_{27}, \end{array}$$

where *rc*_18 _is the binding rate constant from AKT to p53 and [AKT] is the concentration of the AKT molecule. Also, *rc*_20 _represents the binding rate constant for HER2 to AKT. Similarly, for p53, variables in the corresponding equation incorporate information for the molecular concentrations and the binding rate constants for AKT and p53.

In our study, the ODEs were solved using the standard Matlab library function *ode45*. This function can interpret the differential equations in the form of rate reactions. The *ode45 *accepts the initial values of the molecules and the time duration in the form of function parameters [20,21]. Using this procedure, we derived the rate equations and time course data for all twenty-two signalling proteins. The initial concentrations of the signalling molecules were set to dimensionless numeric value of one [22]. Some of the rate constants were taken directly from the literature. These include Casp9 & Casp3, Casp9 & XIAP, Casp8 & BID, Casp3 & XIAP, Casp3 &Casp8, Casp3 & Apop, p53 & PUMA, Casp3 & Casp6 and XIAP & SMAC [4,23,24]. The remaining parameters were generated randomly but we verified them by Partial Least Square Regression (PLSR) test. We also performed sensitivity analysis of the model parameters by varying the model parameters to ensure overall robustness of the model to the small variations of parameters (see result section for PLSR test and sensitivity analysis).

### Genetic algorithm

We applied the Genetic Algorithm to search for the network topologies that fit the real data well, in order to uncover the rewiring inside the tumour cells. There are several optimisation techniques available in the literature which can be used to search for the network topologies. These techniques include, for example, Simulated Annealing, Evolution Strategies, Genetic Algorithm, Evolutionary Programming etc. Simulated Annealing has population size of only one which represents current solution, thus the solution space is not as wide as GA. Similarly, Evolutionary Programming can only use mutation operator. By contrast, Genetic Algorithms have several solutions in the beginning and crossovers can be performed to generate the diversity in the population of network structures. While the probabilistic selection of GA helps us simulate the stochastic biochemical networks in better ways, Evolution Strategies uses deterministic selection [25,26]. After comparing these techniques, we decided to use Genetic Algorithm in our study. As a global search technique, GA is considered to be very robust as it makes very few assumptions about the problem under consideration [27].

The implementation of Genetic Algorithm assumes that the variables from problem can be represented in the form of binary strings typically known as Chromosomes. A feasible solution is also encoded in the form of a chromosome. The initial population for the Genetic Algorithm is a random set of chromosomes. These chromosomes are evaluated using an objective function. The chromosomes representing better solutions are given higher probability to reproduce offspring in next generation. Chromosomes with lower scores are removed from the solution set. The pseudocode for the Genetic algorithm used in our experiments is given in Table 1.

**Table 1:** Genetic Algorithm for finding mutation in apoptotic network.

INPUT: Objective function, Network Topology(NT) in the form of Adjacency vector of size n × n, reaction rate constants vector of size n × n, maximum number of generations Max num gen for the algorithm, biological data measurements
OUTPUT: A vector consisting topology of best uncovered network, score vector S, for such uncovered networks
1. NT_0 _⇐ topology
2. RC_0 _⇐ rate constants
3. Max_num_itr ⇐ total number of iteration allowed
4. S_0 _⇐ 0
5. Derive Population P (randomly generated networks)
6. For max_ num_gen times do:
7. Derive rate equations for each network in P
8. Solve each of the Rate equations
9. Derive numerical solutions (time series data)
10. Compare the simulation results with Yaffe's data using DTW Objective function and calculate the Score
11. Select 50 best score
12. Perform crossover among best selected networks to formulate next generation, total of 100 networks again. The crossover points are selected based on random numbers. The networks to be crossed over are selected randomly.
13. Perform mutation for each bit with the probability of 0.01, for each of the hundred networks.
14. Set new population P = mutated network from step 13
15. Check for the convergence.
16. If network not converged, go to step 5
7. Output solution set.

The network topology is represented by an adjacency matrix. For each edge between two nodes in the network there is a Boolean value of ' 1' in the matrix, and a ' 0' entry represents the absence of connection between the corresponding nodes. The adjacency matrix can be modified in Genetic Algorithm as follows [27]. We initially derive the chromosome (vector of 1 × 484) by concatenating the rows of the adjacency matrix of the network into one binary string. As such, the network topology of 22 nodes is represented as a chromosome i.e. a binary string of length 484. The topology of the basic network *N*_1 _was changed randomly using mutation and crossover. The crossover points were randomly selected uniformly from the range of [1:484]. Generally, the mutation rate for any particular bit is less than 1% [27]. In our implementation the mutation operator was applied to each of the bit in a chromosome (after performing crossover) with a low probability value of 0.01. Once the process of selection of chromosome, recombination and mutation is finished, the population of next generation is evaluated [27].

To compare the time series data, we used Dynamic Time Warping (DTW) [28,29]. DTW is widely used for comparing time series data, due to its strength to capture the variation in data that may vary owing to different speed. Hence, DTW can calculate the optimal match between two temporal data. The objective function of DTW, for comparing two time series datasets *s *and *t *is given as:

$$D\left[i,j\right]=cost+min\left\{\begin{array}{c} D\left[i-1,j\right]\\ D\left[i,j-1\right]\\ D\left[i-1,j-1\right], \end{array}\right)$$

where *cost *= *dist*(*s*[*i*], *t*[*j*]), *D *is a matrix of size [0..*n*, 0..*m*], *D*[0, 0] = 0. Initially each entry of *D*[*i*, 0], for 0 <*i *≤ *n*, and *D*[0, *j*], for 0 <*j *≤ *m*, are set to infinity, *i.e*. a larger value which cannot be part of the data sequences. Here *s*[1..*n*] and *t*[1..*m*] are the two time series representing protein phosphorylation levels over time points to be compared, whereas *s*[*i*] and *t*[*j*] represents the data at time points *i *and *j *respectively. For any two numbers *s*[*i*] and *t*[*j*], *dist*(*s*[*i*], *t*[*j*]) represents the distance between the numbers, e.g. *dist*(*s*[*i*], *t*[*j*]) = |*s*[*i*] −*t*[*j*]|. The objective function aims to measure the goodness of fit between the simulation data and real data.

## Results

### PLSR for simulation data from *N*_1_

**Figure 2:**
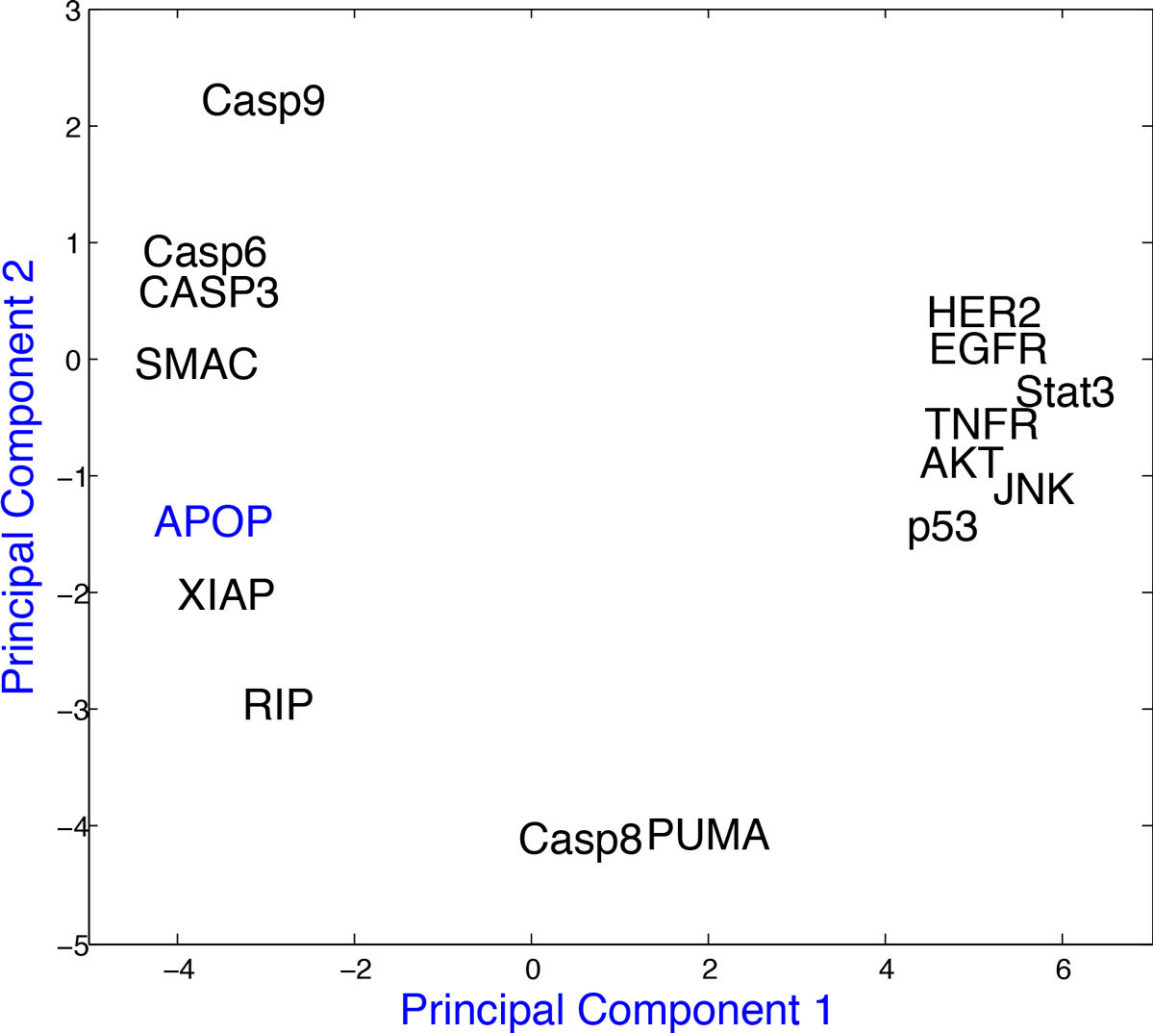
**PLSR plot for simulation data for apoptosis from network *N*_1_**.

We first used Partial Least Squares Regression (PLSR) to test if the simulation data generated using our basic network *N*_1 _were reliable for performing the prediction of network rewiring. PLSR is used for mapping independent variable with dependent variables (e.g. mapping signalling molecules to phenotypic responses) [30]. In a PLSR plot, if a predictor variable is closely associated with the predicted variable, then such a predictor variable influences the predicted variable relatively more strongly than the predictor variable who is far away in the PLSR plot. For reaction parameters, we ensured that the selected parameter values enabled us to associate the important predictor variables like Caspases, XIAP, SMAC etc. closely with the apoptosis. The Partial least Square Regression (PLSR) plot for the simulation data from the basic network from *N*_1_, is given in Figure 2, which PLSR plot shows the close association between apoptosis and several apoptotic proteins. This result is consistent with the results reported by Lee *et al*. [5].

### Inferring the tumorigenic network *N*_2_

**Figure 3:**
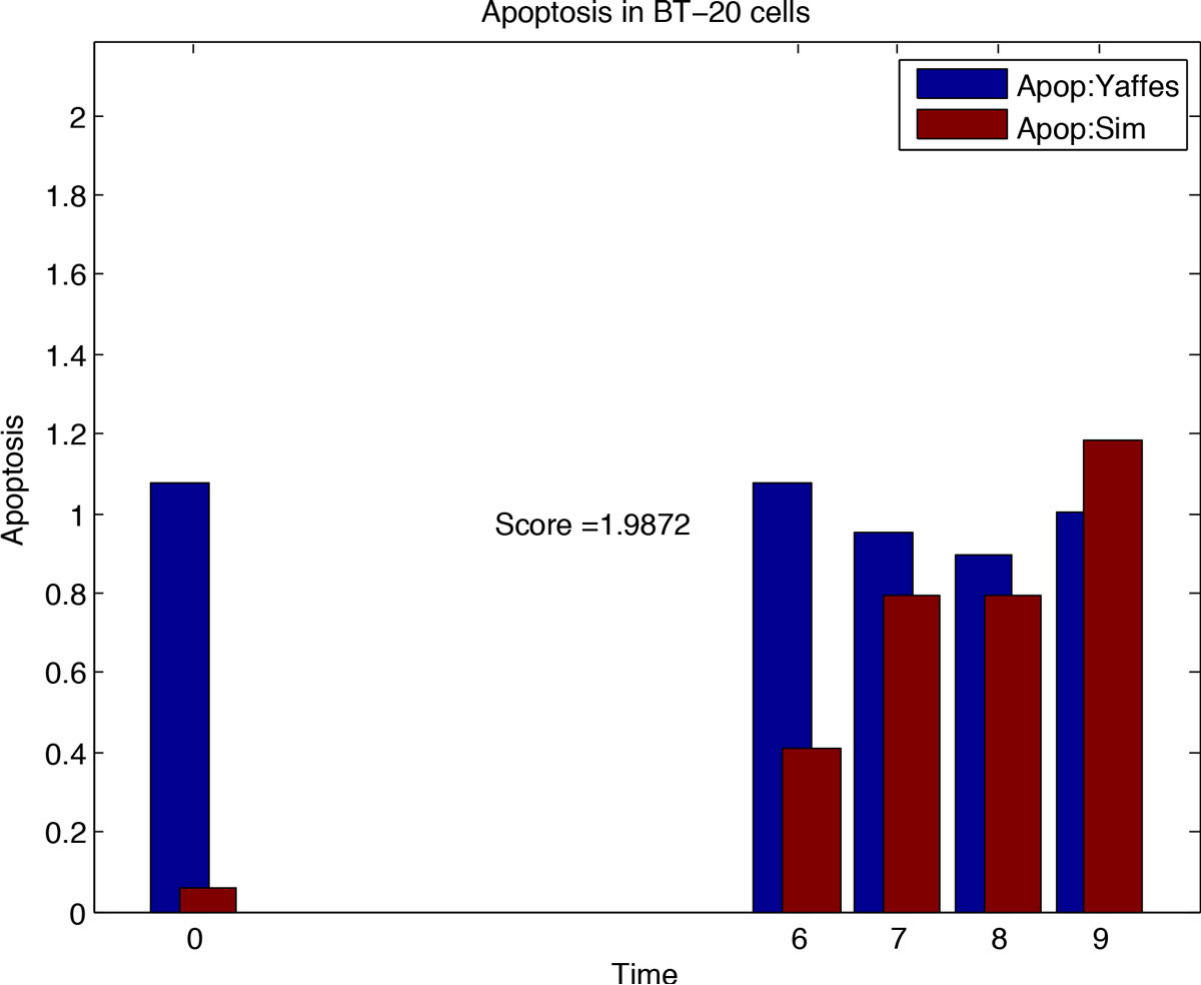
**Simulation results for Genetic Algorithm: fitting experimental data with simulation data**. (**A**) Simulation result for basic apoptotic network, (**B**) Simulation result for DMSO treatment. (**C**) Comparing *N*_2 _based simulation data with ERL-DOX treatments data by correlation of apoptosis rates over time points. (**D**) Simulation result for ERL-DOX treatments.

**Figure 4:**
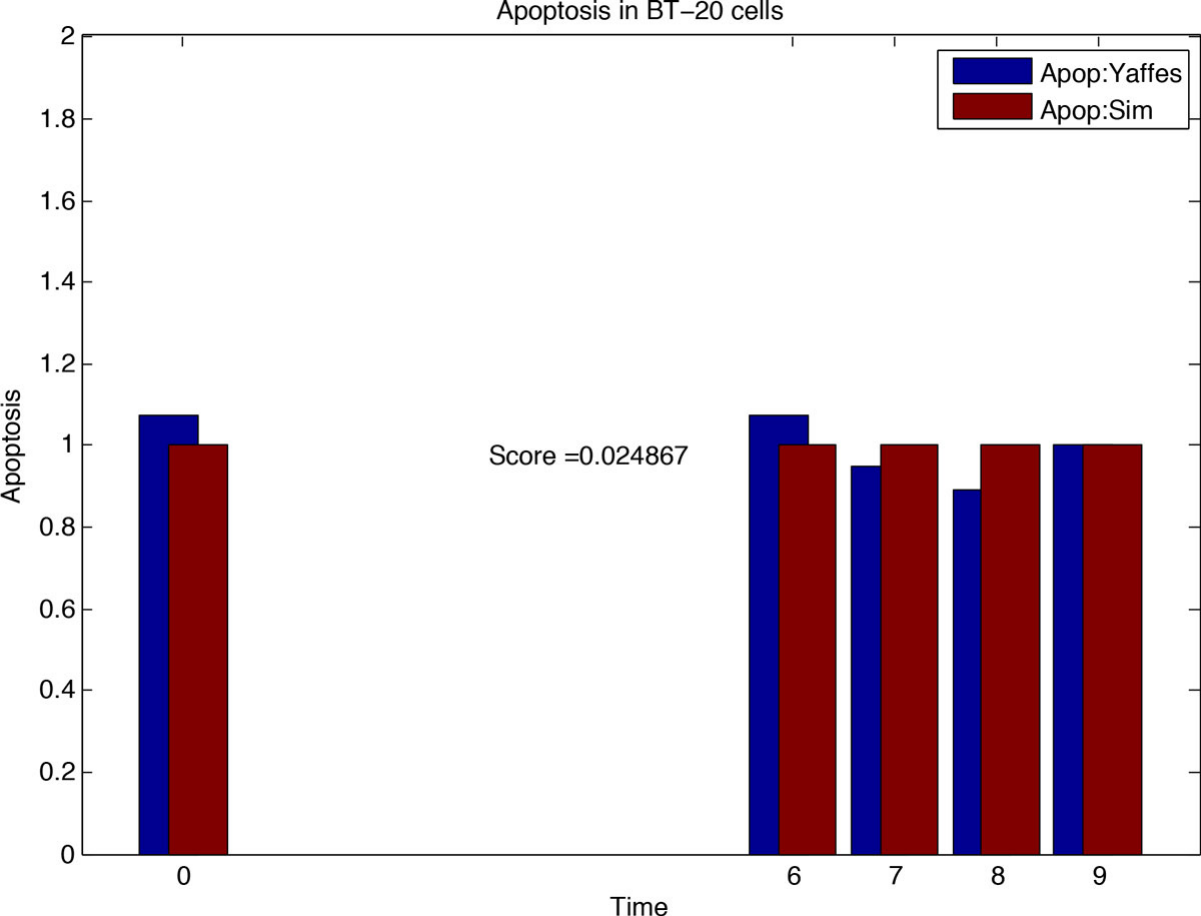


From network *N*_1_, we will infer the rewired network *N*_2 _corresponding to cancer cell lines by fitting simulated data to the cancer specific dataset. To this end, we adopt Genetic Algorithm for multiple generations to search for the most likely network rewiring events and collected 50 rewired networks in each generations along with corresponding scores. The Genetic Algorithm performed rewiring on the basic apoptotic network of Figure 1. The comparison for simulated data from network *N*_1 _and Yaffe's data for control treatments using Genetic Algorithm, before and after rewiring event in network *N*_1_, is shown in Figure 3 and 4.

Due to inherent randomness in Genetic Algorithm, the edges added and deleted in *N*_1 _(while inferring *N*_2_) were different among different run of simulations. Some of the changes in the network were found to be more frequent compared to other. So, just few runs of simulation couldn't be relied upon to find the changes in the *N*_1 _network causing cancer. Therefore, to find consistent (or conserved re-wirings) changes to the network, we performed the simulation for 150 times for inferring *N*_2 _from *N*_1 _and another 150 rounds of simulation for inferring *N*_3 _from *N*_2_. The changes which occurred in the network with the highest frequency were selected to construct *N*_2 _(and subsequently *N*_3_). The most frequently removed edges in the *N*_1 _network while inferring *N*_2 _were SMAC-XIAP, Casp8-Casp3 and p53-PUMA. For 150 rounds of simulations, each of these deletions occurred with a frequency of 146, 143 and 141 respectively. The network edges found to be inserted in the *N*_1 _by the Genetic Algorithm with the highest frequencies were XIAP-Casp8 and TNFR-Stat3, with frequencies of 149 and 146.

We verified the various rewiring events using the existing literature and confirmed the validity for the above mentioned network changes. p53 is a tumour suppressor and controls the regulation of PUMA. PUMA induces apoptotic signals inside the cells. Less activity of PUMA leads to apoptosis deficiency which in turn leads to the increased risk of cancer [31]. We also realised that SMAC down-regulates XIAP and XIAP down-regulates Casp3, a pro-apoptotic protein. So if SMAC is working properly, there will be more cell death leading to less tumorigenic behaviour by cells. But if SMAC is not able to down-regulates XIAP, there will be less apoptosis and so more chances of cancer [2]. We also came across literature evidence highlighting the critical role of Casp8 for affecting breast cancer directly [5,32]. STAT3 interacts with various molecules involved in programmed cell death, regulating their functions including MOMP formation, which leads to the release of the cytochrome c [33]. XIAP inhibits the processing of Casp8. In fact, proteolytic processing of several caspases, including Casp9, is not allowed in the presences of XIAP. The lack of processing of Casp8 and Casp9 allows them to interact with Casp3 and promote apoptosis [34]. TNFR activates the Stat3 signalling. The stimulation of TNFR leads the phosphorylation of the Stat3 and Stat5b and the phosphorylated molecules are trans-located into the nucleus [35].

### Inferring the drug sensitive network *N*_3_

**Figure 5:**
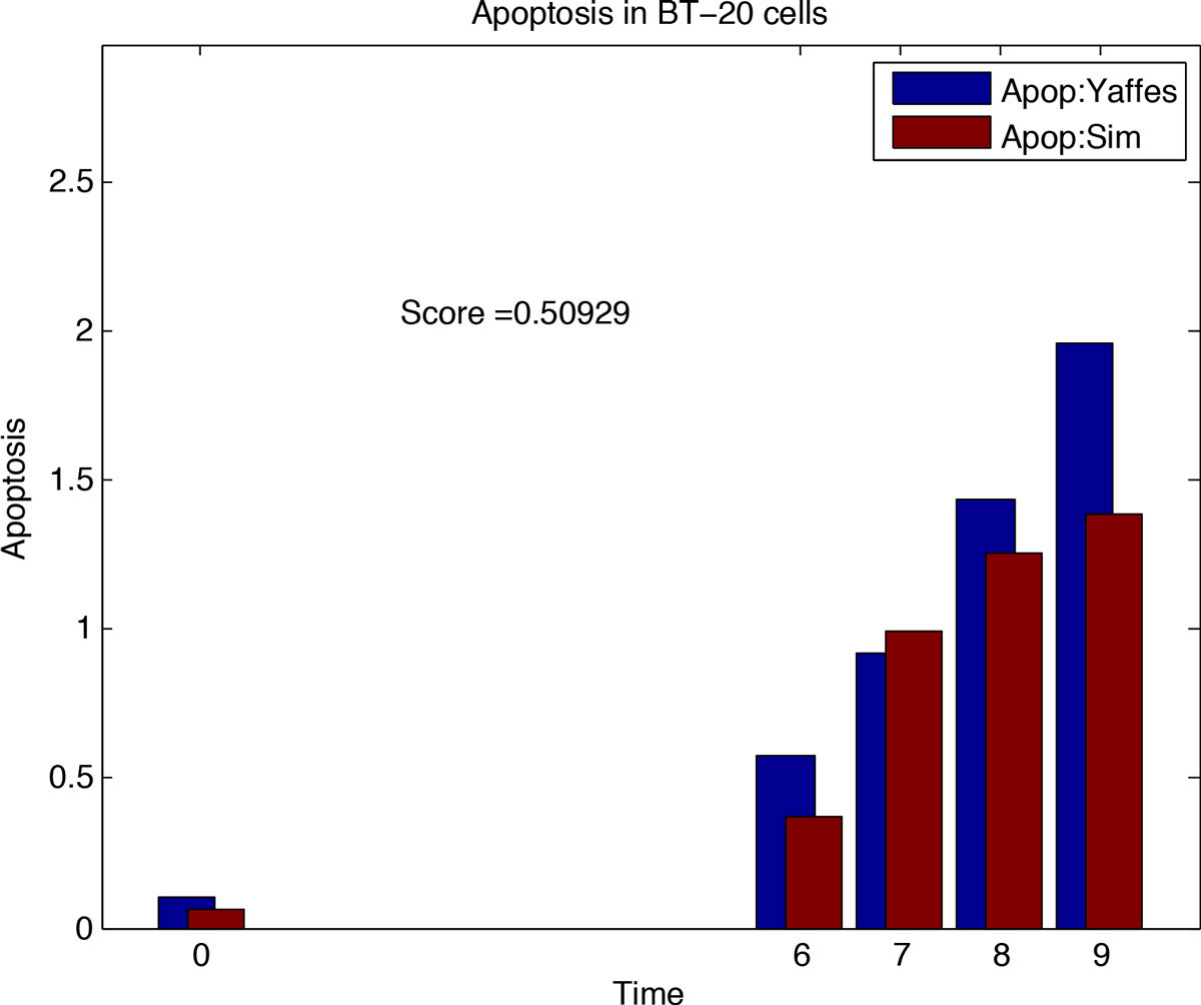


**Figure 6:**
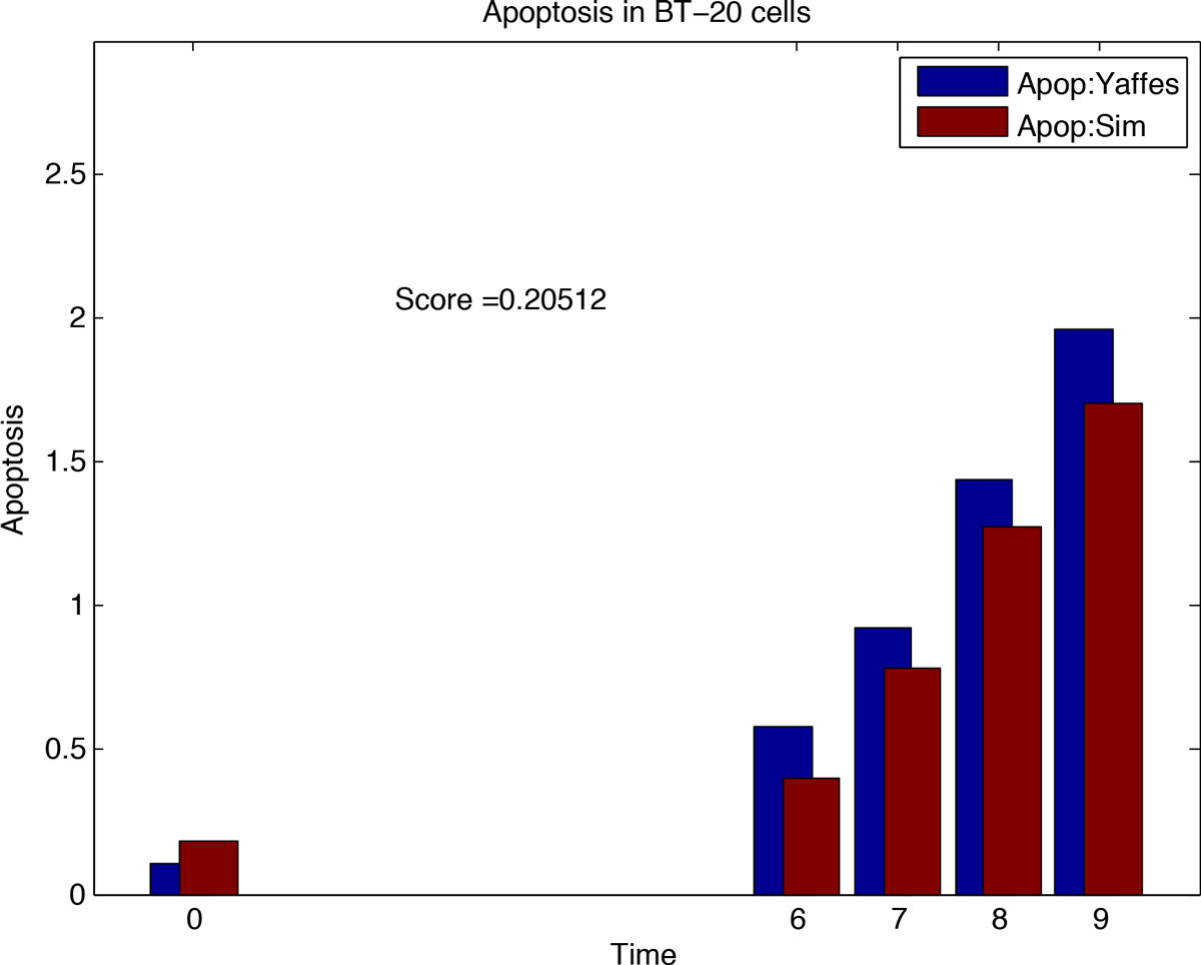


After inferring *N*_2 _from *N*_1_, our aim was to infer the drug sensitive network (*N*_3_), by fitting modified topology of *N*_2 _to the real data of drug treatment. Before predicting rewiring of *N*_2_, we plotted the *N*_2 _based simulation data with the real dataset of signalling and cell fate of cancer cells after treatment with drugs. The plot is shown in Figure 5. We again used Genetic Algorithm to search for the network rewirings that allow better fitting of simulation to the real data. For each run of simulation, we compared the simulation data with the drug sensitive experimental apoptosis data for ERL-DOX treatments. The best network plot, uncovered using Genetic Algorithm, is shown in Figure 6. The edges of *N*_2 _that were most frequently removed were BIM-Casp9 and EGFR-Casp8, deleted 141 and 137 times respectively. The network edges inserted with highest frequencies by the Genetic Algorithm were Casp8-RIP and Casp8-MOMP, inserted 144 and 135 times respectively. These rewiring events have been mentioned in related literature [2,5,17,36].

### Sensitivity analysis of the model

To analyse the behaviour of our model with respect to the fluctuations in model parameters, we verified our model by performing sensitivity analysis for the rate constants. We initially tested apoptosis level for each of the molecules by reducing the parameter by 30%. Then we increased the parameter by 30%. Among all the parameters, "Casp3-Casp8" and "Casp3-Apop", were found to be very sensitive, affecting the apoptosis level to the maximum. We found that the level of apoptosis for most of the parameters changed only slightly, within +/-5%, except for the most sensitive parameters, "Casp3-Casp8" and "Casp3-Apop", which affected the level of apoptosis by +/-20%.

To test the robustness of our model, we also performed the perturbation test. We changed the initial concentration of each of the molecules and identified the most sensitive parameters of the model. The apoptosis level was predicted for each of the molecule whose initial concentration was reduced by 30%. Then, for each molecule we increased the initial concentration by 30%. We found that the level of apoptosis for most of the initial concentrations was stable, except for the concentration of the molecules with parameters of "Casp3-Casp8" and "Casp3-Apop". This suggests an overall robustness of our model.

## Discussion and conclusion

In our proposed study, we used Genetic Algorithm to infer rewiring of the apoptotic network. The most frequent rewiring events are selected, and most of them were found to be consistent with existing literature. However, using Genetic Algorithm we may not find a globally optimal solution, as some of the network rewiring events might have been missed by the Genetic Algorithm based searching. So far we have included only 18 out of 32 proteins in the Yaffe's data. A network with more signalling data may help us understand the pathway changes in more comprehensive ways. In future, with expanded apoptosis network and more proteins, we would be able to detect more network alterations through comparisons between simulation data and experimental data. Also, beside Genetic Algorithm we plan to use other computational techniques (e.g. Simulated Annealing). In this paper Yaffes data set has been used. Our next goal is to construct a larger network model for cancer cell phenotypic responses which can simulate observations for some other datasets taken from multiple sources.

## Competing interests

The authors declare that they have no competing interests.

## Authors' contributions

SKM and JZ conceived and designed the computational model and SKM performed the *in silico *experiments, and drafted the manuscript. SSB gave suggestions for the experiments and assisted in the formulation of the model. HEC participated in discussions and provided feedbacks for the experiments. FZ assisted in formulation of experiments and proof-reading of the manuscript. JZ supervised and reviewed the manuscript. All authors read and approved the final manuscript.
